# The Survival Rate and Its Influencing Factors of Modular Endoprosthetic Replacement With Uncemented Stem for the Proximal Femur After Primary Tumor Resection

**DOI:** 10.1111/os.70146

**Published:** 2025-09-04

**Authors:** Zhuangzhuang Li, Yi Luo, Yitian Wang, Taojun Gong, Xuanhong He, Yong Zhou, Minxun Lu, Li Min, Chongqi Tu

**Affiliations:** ^1^ Department of Orthopedics, Orthopedic Research Institute West China Hospital, Sichuan University Chengdu China; ^2^ Model Worker and Craftsman Talent Innovation Workshop of Sichuan Province Chengdu China

**Keywords:** aseptic loosening, implant survivorship, modular uncemented endoprosthesis, primary bone tumors, proximal femoral replacement

## Abstract

**Objective:**

Durable and biologically integrated fixation is critical for long‐term implant survival in patients with primary bone tumors. However, limited evidence exists regarding the long‐term outcomes of uncemented stem designs in this population. Specifically, we investigated: (1) the long‐term patient and implant survivorship rates; (2) the influence of factors such as resection length and patient age on implant survival; and (3) the incidence and types of complications, particularly those requiring implant removal or revision.

**Methods:**

We retrospectively reviewed 76 patients (49 males, 27 females; mean age 41 years, range 14–78 years) who underwent proximal femoral replacement with a modular uncemented endoprosthesis between 2015 and 2022. The mean follow‐up was 63.4 months (median: 60.5 months; range: 12–104 months). Functional outcomes were assessed using the Musculoskeletal Tumor Society (MSTS) score, while complications were classified based on the Henderson et al. system. Survivorship analyses were conducted using Kaplan–Meier methods.

**Results:**

The 5‐year patient survival rate was 88.0%, and the 5‐year implant survival rate was 90.4%. The mean MSTS score at final follow‐up was 25.6 (range 16–30), with 87.7% of patients achieving good to excellent functional outcomes. Younger patients (< 30 years) exhibited poorer implant survival, while resection length did not significantly impact outcomes. Complications occurred in 18.4% of cases, categorized into type 1 (soft tissue‐related, *n* = 3), type 2 (aseptic loosening, *n* = 2), type 3 (structural failure, *n* = 0), type 4 (infection, *n* = 3), type 5 (tumor recurrence, *n* = 3), and three cases of acetabular failure.

**Conclusions:**

Modular uncemented endoprostheses for proximal femoral replacement demonstrated promising survivorship and functional outcomes in patients with primary bone tumors. The low rate of aseptic loosening highlights the benefits of uncemented stem designs. However, younger age remains a risk factor for reduced implant longevity.

## Graphical abstract

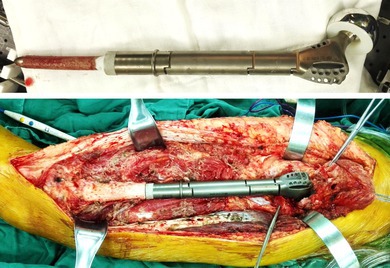

## 1 Introduction

The proximal femur is a common anatomical location for primary malignant and benign lesions as well as metastatic disease [1, 2, 3, 4, 5]. With the advances in neo‐adjuvant chemotherapy and surgical techniques, limb salvage surgery has become the preferred treatment modality in the majority of cases [5, 6, 7]. Among reconstruction methods, modular endoprosthetic replacement has gained widespread acceptance due to its advantages, including cost‐effectiveness and broad availability [1, 8, 9]. Research on modular endoprostheses has demonstrated acceptable outcomes in terms of durability and functional recovery [10, 11]. However, a significant proportion of studies include relatively a large number of metastatic tumor patients [12, 13], whose relatively short survival time may confound the understanding of long‐term outcomes.

For patients with primary bone tumors who typically have longer life expectancies, durable fixation of the endoprosthesis to the diaphyseal femoral shaft is challenging [14]. Cemented endoprostheses have been widely adopted due to their ability to provide immediate and reliable fixation. Their use is technically straightforward and allows for early weight‐bearing in the postoperative period. However, several limitations have emerged with long‐term follow‐up. The exothermic reaction during PMMA polymerization can lead to thermal necrosis of the surrounding bone, potentially compromising the initial bone–implant interface [15]. More importantly, the long‐term fixation stability is undermined by a relatively high rate of aseptic loosening, partly due to stress shielding and the inability of the cement interface to remodel in response to physiological loading [16]. Compared with cemented endoprostheses, uncemented endoprosthesis uses the press‐fit technique for fixation, with a cylindrical stem fitting tightly into the prepared femoral cavity to provide initial stability and subsequent osseointegration. Theoretically, uncemented endoprostheses have the potential to achieve biological and possibly permanent fixation [17, 18]. Therefore, the application of uncemented endoprosthesis has recently gained favor in proximal femoral replacement [19, 20]. However, the long‐term efficacy and safety of uncemented modular systems in proximal femur replacement remain areas of ongoing research.

This study aimed to evaluate the survivorship and functional outcomes of proximal femoral replacement using modular uncemented endoprostheses in patients with primary bone tumors. Specifically, we investigated: (1) the long‐term patient and implant survivorship rates; (2) the influence of factors such as resection length and patient age on implant survival; and (3) the incidence and types of complications, particularly those requiring implant removal or revision.

## 2 Patients and Methods

### 2.1 Patients

This retrospective cohort study was conducted after approval by the Ethics Committee of West China Hospital, Sichuan University (Approval No. 2022‐43). The study included a consecutive series of patients who underwent modular uncemented endoprosthetic replacement between 2015 and 2022. Patients were included based on the following criteria: (1) histologically confirmed primary bone tumor or soft tissue tumor invading the bone, (2) treatment with modular endoprosthetic replacement with an uncemented stem following tumor resection, (3) a minimum follow‐up period of 2 years or experienced the complication within 2 years, and (4) complete follow‐up information. Exclusion criteria were (1) prior history of hip arthroplasty, (2) metastatic disease, (3) use of endoprosthesis allograft composite, or (4) use of a 3D‐printed custom‐made uncemented stem in metaphysis [21].

A total of 76 patients were included in this study (Table 1), with a mean follow‐up of 63.4 months (median: 60.5 months; range: 12–104 months). The mean age at surgery was 39.7 years (range: 14–78 years), and the cohort consisted of 49 male patients and 27 female patients. The primary diagnoses were 35 cases of osteosarcoma, 21 cases of chondrosarcoma, 5 cases of giant cell tumor of bone (GCTB), 4 cases of Ewing sarcoma, and 11 cases of soft tissue sarcoma (STS). Neoadjuvant chemotherapy was administered in 49 patients (64.5%), with the distribution as follows: all 35 osteosarcoma cases (100%), all 4 Ewing sarcoma cases (100%), 8 of 11 soft tissue sarcoma cases (72.7%), and 2 of 21 chondrosarcoma cases (9.5%). The 2 chondrosarcoma patients who received chemotherapy were diagnosed with dedifferentiated chondrosarcoma, a subtype known to respond to chemotherapy. Radiotherapy was used in 4 patients (5.3%), including 3 patients with STS and 1 patient with Ewing sarcoma. Among the 5 cases of GCTB, all were classified as Campanacci grade 3 lesions, characterized by aggressive behavior with cortical destruction and soft tissue extension. These patients underwent bony resection due to the high risk of local recurrence and the need for safe surgical margins to achieve disease control. Bony resection was performed in STS cases where the tumor directly invaded the bone or was in close proximity to critical structures, making limb‐sparing surgery feasible only with bone resection.

**TABLE 1:** Clinical characteristics of the patients who received modular uncemented proximal femur replacement.

Characteristic	Data, *n* (%)
Sex	
Male	49 (64.5)
Female	27 (35.5)
Age	
Young: < 30 years	23 (30.2)
Middle‐aged: ≥ 30 ≤ 60 years	42 (55.3)
Old‐aged: > 60 years	11 (14.5)
Diagnosis	
Osteosarcoma	35 (46.0)
Chondrosarcoma	21 (27.6)
Giant cell tumor	5 (6.6)
Ewing's sarcoma	4 (5.3)
Soft tissue sarcoma	11 (14.5)
Resection/total length	
< 40%	38 (50.0)
≥ 40 ≤ 60%	27 (35.5)
> 60%	11 (14.5)
Chemotherapy	
Yes	49 (64.5)
No	27 (35.5)
Radiotherapy	
Yes	4 (5.3)
No	72 (94.7)

### 2.2 Features of Modular Uncemented Endoprosthesis

**FIGURE 1:**
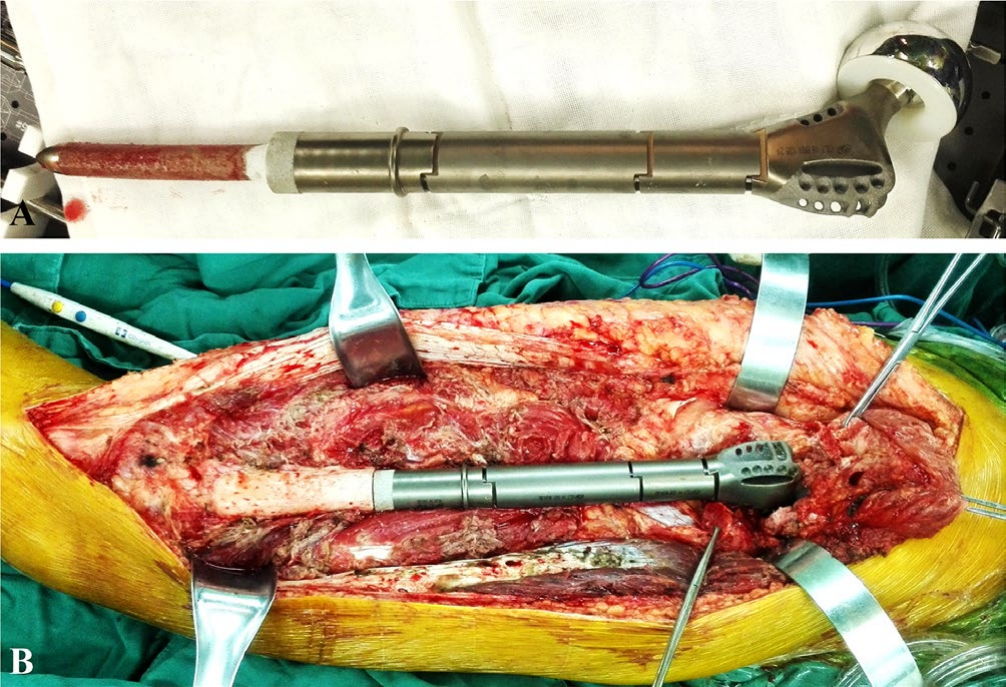
(A) Modular endoprosthesis for proximal femoral replacement, comprising five modular components: the uncemented stem, diaphysis segment, proximal femoral segment, femoral head, and acetabular component. (B) Intraoperative image showing the implantation of the modular endoprosthesis system.

All endoprosthetic systems were manufactured by Beijing Chunlizhengda Medical Instruments (Tongzhou, Beijing, China). The endoprosthesis for proximal femoral replacement included five modular components: the uncemented stem, diaphysis segment, proximal femoral segment, femoral head, and acetabular component (Figure 1). The uncemented stem was designed with a short, curved geometry, crafted using forging technology and composed of a titanium alloy (Ti6Al4V) with a sprayed titanium and hydroxyapatite (HA) coating. The stem measured 110 mm in length, including a 10 mm non‐pressurized tip, and featured a taper of 0.1. It was available in diameters ranging from 10 to 18 mm and a curvature degree of 3°. Symmetrical medial and lateral flanges at the stem's base prevented rotation and ensured stability. The proximal femoral segment was configured with an enlarged protuberance to mimic the natural shape of the femur. To preserve tendinous attachments to the greater and lesser trochanters, the endoprosthesis was designed with multiple suture holes and grooves at the proximal segment. These features allowed for suturing of the tendons and soft tissues to the prosthesis during surgery. The femoral head component featured a dual‐mobility design to reduce the risk of dislocation [8, 22], ensuring enhanced stability and range of motion for the joint.

### 2.3 Surgical Technique

The patient was placed in a lateral decubitus position. Carefully dissect through the layers of skin, subcutaneous tissue, and muscles to reach the tumor site. After clear visualization of the tumor, neurovascular structures, and adjacent anatomical landmarks, the tumor was gently dissected from adjacent tissues. An osteotomy mark was made following careful measurement of osteotomy distance according to the preoperative plan. After that, an appropriate osteotomy was performed to remove the affected portion of the proximal femur. The intramedullary canal was sequentially reamed using a technique where the final reamer size was 1 mm smaller than the diameter of the chosen uncemented stem. In cases where the bone quality was compromised, prophylactic cerclage cables were placed around the femur to reinforce the cortical integrity and prevent fractures. Then, the definitive uncemented stem was inserted with the correct orientation and alignment. The selection of proximal femoral endoprosthetic reconstruction bearing either hemiarthroplasty or total arthroplasty was made on a case‐by‐case basis. In cases where the greater trochanter was preserved, it was secured onto the implant using a combination of cerclage wires and non‐absorbable sutures. The trochanteric fragment was carefully positioned to restore the anatomical insertion of the abductor muscles, and the fixation was reinforced to withstand postoperative tension during rehabilitation. When the greater trochanter could not be preserved, the abductor tendons were directly reattached to the implant. The tendons were tensioned under slight abduction to ensure optimal biomechanical function.

### 2.4 Outcome Assessment

Postoperatively, patients were kept non‐weight‐bearing for the first 6 weeks to allow for initial osseointegration and soft tissue healing. During this period, passive range‐of‐motion exercises were initiated to prevent joint stiffness. After 6 weeks, partial weight‐bearing was gradually introduced, progressing to full weight‐bearing by 12 weeks postoperatively, depending on radiographic evidence of bone healing and patient tolerance. Regular follow‐up assessments were conducted at predefined intervals, including monthly evaluations for the first 3 months and quarterly visits thereafter. Each follow‐up included a comprehensive functional assessment and radiographic evaluation to monitor implant stability and patient recovery. Functional assessment was performed using the Musculoskeletal Tumor Society (MSTS) score [23]. Regarding radiographic evaluation, standard X‐rays and digital Tomosynthesis‐Shimadzu Metal Artifact Reduction Technology (T‐SMART) images were collected and evaluated independently by two experienced musculoskeletal oncology surgeons. T‐SMART has been shown to enhance visualization of periprosthetic bone in the presence of metal implants by reducing artifacts and improving contrast [24]. The assessment protocol for osseointegration was adapted from previously validated methods, including our own published research [25]. The femur was divided into four anatomical zones on both anteroposterior and lateral views (anterior, posterior, medial, and lateral). Bone ingrowth was defined by the presence of continuous trabecular structures directly contacting the implant surface. Effective osseointegration was considered present if trabecular continuity extended ≥ 2.5 cm in at least two of the four zones. Implant instability—suggestive of aseptic loosening—was defined as a stem axis deviation ≥ 1° or a lateral/vertical migration ≥ 1 mm on serial imaging.

The primary outcome measures were (1) patient and endoprosthesis survivorship, defined as the time from surgery to either patient death or partial or total revision of the implant; and (2) functional outcomes, as assessed by the MSTS scoring system at the final follow‐up. Secondary outcomes included postoperative complications, recorded and classified into five types according to Henderson et al. [17]. Particularly, patients who encountered severe pain with radiographic signs of acetabular wear and required conversion of a hemiarthroplasty to total arthroplasty were considered as “acetabular failure”.

### 2.5 Statistical Analyses

Statistical analyses were performed using SPSS Statistics software version 26.0 (IBM Corp., Armonk, NY, USA) and Origin software version 2021 (OriginLab Corp., Northampton, MA, USA). Descriptive statistics, including means, medians, and percentages, were calculated. Kaplan–Meier survival analysis was conducted to generate survival curves, and the log‐rank test was used to compare endoprosthesis survival. A *p*‐value of less than 0.05 was considered statistically significant.

## 3 Results

### 3.1 Patient Survival

**FIGURE 2:**
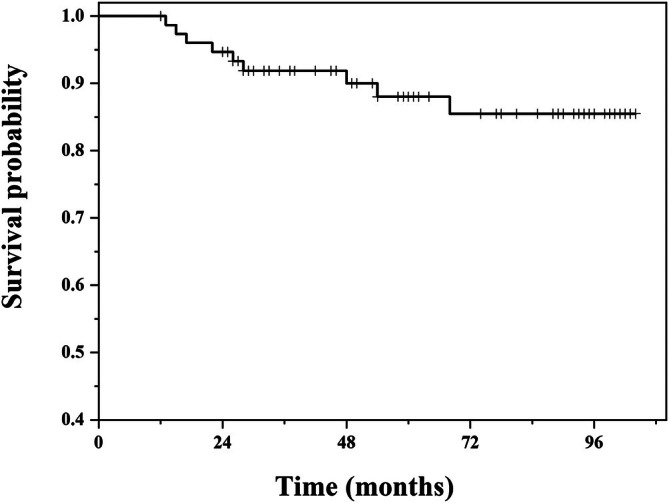
Kaplan–Meier survival curve of patients who underwent proximal femoral replacement, showing survival rates of 88.0% at 5 years and 85.5% at 7.5 years.

Nine of the 76 patients (11.8%) died due to pulmonary metastases, with a mean time of 15.5 months (median: 26 months; range, 13–68 months). Among them, eight cases were osteosarcoma, and one was Ewing's sarcoma. The patient survival rates at 5 and 7.5 years were 88.0% and 85.5%, respectively (Figure 2).

### 3.2 Complications

Among the 76 proximal femur reconstructions, 62 were hemiarthroplasty replacements (81.6%), while 14 were total hip arthroplasty replacements (18.4%). During the follow‐up period, a total of 14 patients (18.4%) experienced complications, categorized into type 1 (soft tissue‐related, *n* = 3), type 2 (aseptic loosening, *n* = 2), type 3 (structural failure, *n* = 0), type 4 (infection, *n* = 3), type 5 (tumor recurrence, *n* = 3), and three cases of acetabular failure.

#### 3.2.1 Type 1 (Soft Tissue‐Related)

In detail, dislocation occurred in three patients (4.0%) at a median of 3 months postoperatively (range, 2–5 months). Among these cases, the initial reconstructions included two bipolar hemiarthroplasties and one total hip arthroplasty. The dislocations were managed successfully with open reduction in two patients and closed reduction in one patient. All patients retained the original endoprostheses, and no further dislocations were observed during follow‐up.

#### 3.2.2 Type 2 (Aseptic Loosening)

**FIGURE 3:**
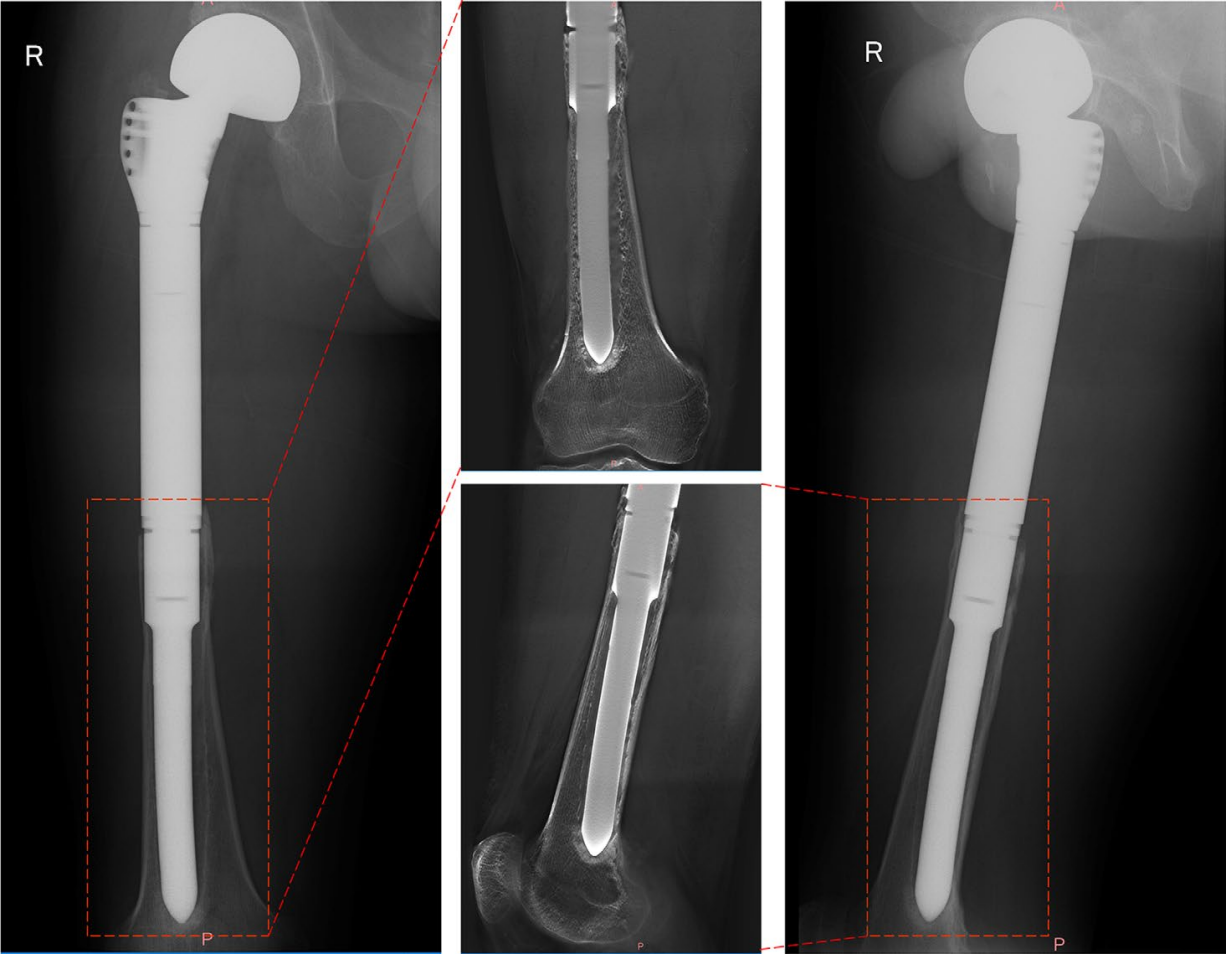
An aseptic loosening stem in a 25‐year‐old male patient, 18 months after implantation. Radiographic evaluations on anteroposterior and lateral views, combined with Tomosynthesis‐Shimadzu Metal Artifact Reduction Technology (T‐SMART).

**FIGURE 4:**
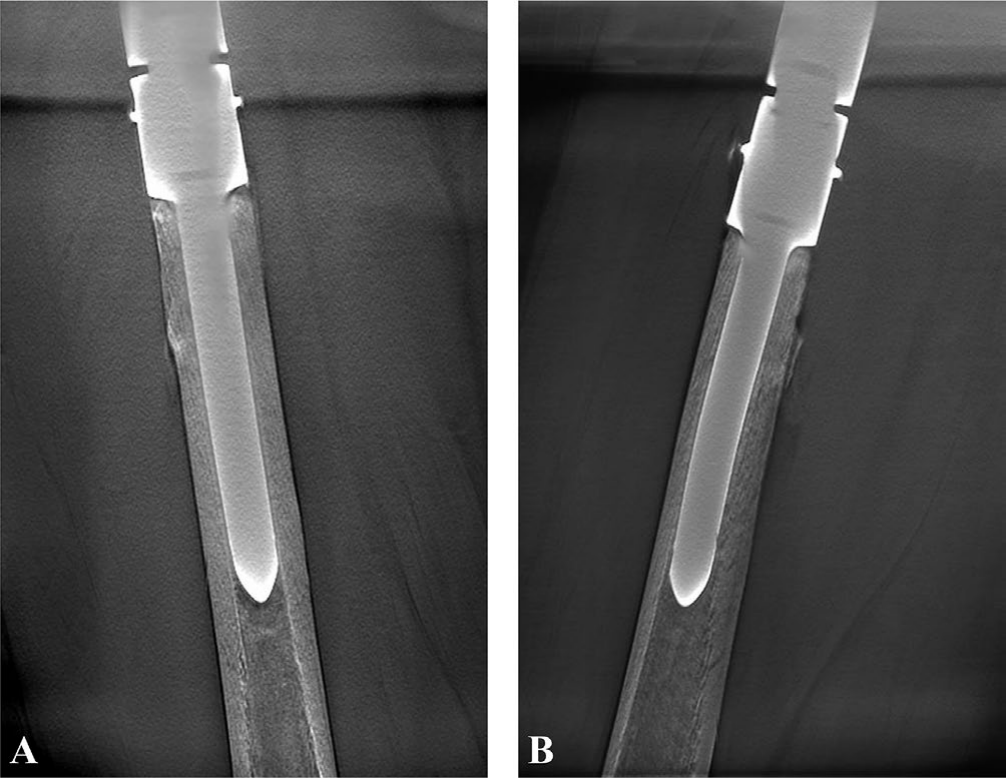
(A) Anteroposterior and (B) lateral views using Tomosynthesis‐Shimadzu Metal Artifact Reduction Technology (T‐SMART) demonstrate excellent osseointegration between the bone and the stem in a 38‐year‐old female patient at 49 months postoperatively.

Aseptic loosening was observed in two patients (2.6%) within 2 years postoperatively at 10 and 18 months, respectively (Figure 3). Both cases underwent thorough diagnostic evaluation, including serum inflammatory markers (CRP and ESR) and intraoperative tissue cultures. The cultures were negative, and there was no clinical or laboratory evidence of infection, confirming the diagnosis of aseptic loosening. Two cases were managed with revision surgery, involving replacement of the loosened stem to restore stability. Following the revisions, no further episodes of aseptic loosening were reported during subsequent follow‐up. The remaining patients demonstrated good osseointegration (Figure 4), with stable fixation of the endoprosthesis. Throughout the follow‐up period, no evidence of structural failure was observed, such as periprosthetic fracture, stem breakage, or endoprosthesis breakage.

#### 3.2.3 Type 4 (Infection)

Delayed wound healing occurred in one patient 6 months after surgery. The patient was managed with revision surgery involving wound expansion and vacuum‐assisted closure (VAC) therapy. The initial endoprosthesis was retained, and no further complications related to the implant were observed during follow‐up. This case was classified as an infection‐related complication due to the chronic nature of the wound and the need for surgical intervention, consistent with infectious disease (ID) guidelines. Additionally, deep infections occurred in two patients (2.6%) at 4 months and 6 months following the primary surgery. Notably, neither patient had undergone radiotherapy. Both cases were managed with a two‐stage revision. In the first stage, the modular components were removed, and an antibiotic‐loaded cement spacer was implanted. Cultures identified *Staphylococcus aureus* in one patient and Coagulase‐negative Staphylococcus in the other. Both patients received targeted intravenous antibiotics for 6 weeks, followed by oral antibiotics for an additional 6 weeks. The second‐stage revision, involving implantation of a new modular endoprosthesis, was performed following confirmation of infection eradication through aspiration and normalization of inflammatory markers.

#### 3.2.4 Type 5 (Tumor Recurrence)

Tumor recurrence was observed in three patients (4.0%) at a median of 29 months postoperatively (range, 12–33 months). Among these, two cases were osteosarcoma, and one was soft tissue sarcoma (fibrosarcoma). All three recurrences were intra‐articular, involving the hip joint and adjacent soft tissues, which necessitated hemipelvectomy amputation as definitive treatment to achieve wide surgical margins and local disease control. The remaining 73 patients (96.0%) achieved limb salvage.

#### 3.2.5 Acetabular Failure

Acetabular failure was observed in three patients (4.0%) at 49 months, 53 months, and 76 months post‐surgery. All patients presented with severe hip pain accompanied by radiological signs of wear. This was managed by converting the hemiarthroplasty to a total hip arthroplasty, successfully restoring joint function.

### 3.3 Endoprosthesis Survival

**FIGURE 5:**
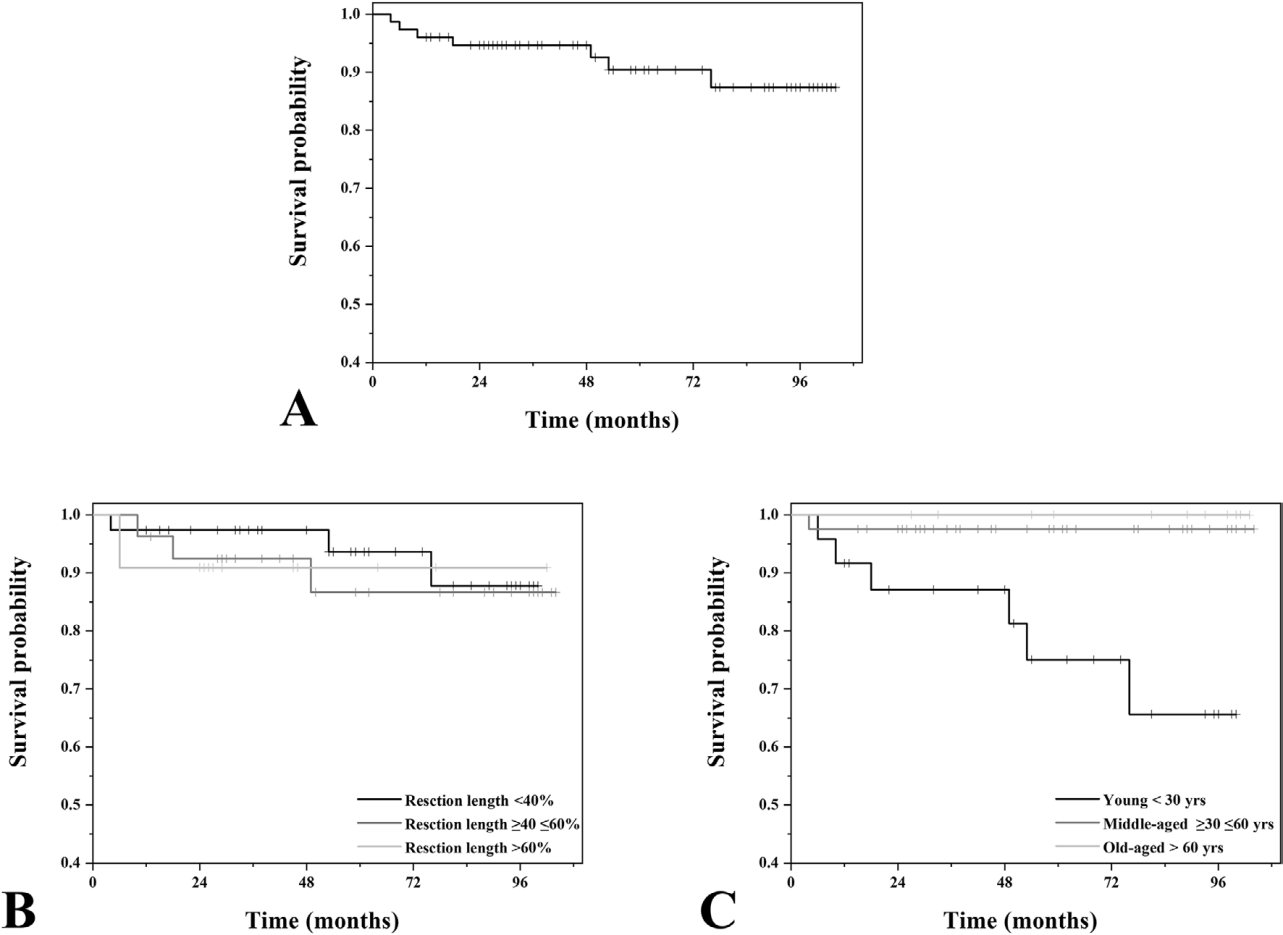
(A) Kaplan–Meier survival curve showing endoprosthesis survival rates of 90.4% at 5 years and 87.4% at 7.5 years. (B) Comparison of endoprosthesis survival based on resection length revealed no statistically significant difference (*p* = 0.86). (C) Age‐stratified analysis demonstrated a significant association between age and survival, with patients younger than 30 years showing a trend toward poorer prosthesis survival (*p* = 0.01).

So collectively, seven of the 76 patients (9.2%) underwent revision of their implants with a mean interval of 30.9 months (median: 18.0 months; range 4–76 months). There were two cases of stem loosening, two of deep infection, and three of acetabular failure (Table 2). The endoprosthesis survival rates were 90.4% at 5 years and 87.4% at 7.5 years (Figure 5A). No statistically significant difference in survival was observed with respect to resection length (*p* = 0.86), while age stratification (*p* = 0.01) was significantly associated with survival (Figure 5B,C). Patients younger than 30 years demonstrated a trend toward poorer implant survival, indicating that younger age may be a risk factor for reduced endoprosthesis longevity.

**TABLE 2:** Patients requiring revision of modular component following primary proximal femoral replacement.

Patients	Age, years	Resection length, %	Index diagnosis	Cause of revision	Time to revision, months
1	24	23.1	Chondrosarcoma	Acetabular failure	76
2	21	47.5	Osteosarcoma	Stem loosening	18
3	13	42.1	Osteosarcoma	Stem loosening	10
4	14	60.5	Osteosarcoma	Deep infection	6
5	27	41	Osteosarcoma	Acetabular failure	49
6	37	23.1	Soft tissue sarcoma	Deep infection	4
7	29	30.3	Chondrosarcoma	Acetabular failure	53

### 3.4 Functional Outcomes

Of the 73 patients with limb salvage, the mean MSTS score at the last follow‐up was 25.6 (range, 16–30), reflecting overall satisfactory functional outcomes. Specifically, 27.4% (*n* = 20) of patients achieved excellent function, 60.3% (*n* = 44) had good function, 12.3% (*n* = 9) had fair function, and none were classified as having poor function.

## 4 Discussion

Proximal femoral endoprosthetic replacement is a well‐established and effective approach for restoring function in patients with malignant bone tumors and select non‐neoplastic conditions of the proximal femur [13, 26, 27]. While custom‐made endoprostheses were initially the standard [28, 29], modular endoprostheses have gained preference due to their advantages, including enhanced adaptability and cost‐effectiveness. Despite their widespread adoption, patients with primary bone tumors present unique challenges for modular endoprosthetic replacement. These individuals require endoprostheses with exceptional durability and long‐term performance to meet the demands of prolonged post‐treatment survival and functional restoration. This study examines whether modular endoprostheses featuring uncemented stems can address the needs of patients following the resection of primary tumors, with a focus on survivorship, functional outcomes, and complications.

### 4.1 Patient Survival and Functional Outcomes

This study demonstrated a 5‐year overall survival rate of 88.0% for patients with primary bone tumors undergoing proximal femoral replacement. This survival rate is significantly higher than the rates (16%–58% at 5 year) reported for patients with metastatic disease or mixed cohorts [13, 30, 31, 32]. Additionally, the inclusion of patients with benign tumors requiring proximal femur replacement may contribute to the higher survival rate in this study.

Limb salvage was achieved in 96.0% of patients in our cohort, with 3 patients (4.0%) undergoing amputation due to tumor recurrence. Bernthal et al. reported a low amputation rate of 1.2% in their cohort, which included metastatic patients [11]. This likely reflects a more conservative treatment strategy where limb salvage and quality of life are prioritized over aggressive interventions. The amputation rate in the present study is comparable to the findings of Chandrasekar et al., who reported an overall limb salvage rate of 96% with a 4% amputation rate in their cohort of 100 patients [33]. The causes of amputation in their study included two tumor recurrences and infections, respectively. In our study, the MSTS score among the 96.0% of patients who achieved limb salvage was 25.6. This aligns with the range of MSTS scores reported in the literature for proximal femoral replacements, which typically falls between 21 and 26.2 [11, 12, 31]. These results demonstrate that modular endoprostheses with cemented or cemented stem fixation are effective in restoring function, as the importance of reconstructing the abductor mechanism and the use of dual‐mobility femoral heads are consistently emphasized to achieve favorable functional outcomes [34].

### 4.2 Endoprosthesis Survival

The 5‐year endoprosthesis survival rate in this study was 90.4%, highlighting the promising performance of uncemented endoprostheses compared to those reported in the literature [11, 12, 30, 33, 35] (Table 3). For instance, Chandrasekar et al. evaluated the outcomes of METS cemented endoprostheses in a cohort of 100 patients, including 35 primary bone tumors and 65 metastatic cases, and reported a comparable 5‐year survival rate of 90.7% [33]. Similarly, Guzik (2018) documented a 5‐year survival rate of 83.1% for cemented modular endoprostheses in 100 patients with metastatic proximal femoral tumors [35]. On the other hand, Kim et al. analyzed outcomes for a mixed cohort of 68 patients who underwent proximal femoral replacement using modular bipolar endoprostheses, with a predominant focus on uncemented designs (50 uncemented vs. 18 cemented) [30]. They reported an overall survival rate of 83.9% at 5 years, with cemented fixation showing a tendency toward poorer implant survival.

**TABLE 3:** A comparison of endoprosthesis survival and complications of the published series of modular endoprosthetic replacement following tumor resection.

Study (Year)	Case No.	Diagnosis	Follow‐up (months)	Stem fixation	Survival (5 years)	Aseptic loosening	Infection	Acetabular wear
Chandrasekar (2009) [28]	100	Primary: *n* = 35	24.6 (0–60)	Cemented: *n* = 100	90.7%*	1%	6%	5%
		Metastatic: *n* = 65						
Bernthal (2010) [9]	86	Primary: *n* = 67	64.4 (3–291)	Cemented: *n* = 86	93%	4.7%	1.2%	5.8%
		Metastatic: *n* = 19						
Guzik (2018) [30]	100	Metastatic: *n* = 100	15.9 (0–77)	Cemented: *n* = 100	83.1%	0%	6%	0%
Kim (2021) [25]	68	Primary: *n* = 55	55.6 (6–172)	Cemented: *n* = 18	83.9%	3%	4%	4.4%
		Metastatic: *n* = 13		Uncemented: *n* = 50				
Abou Senna (2022) [10]	60	Primary: *n* = 44	58 (26–167)	Cemented: *n* = 55	NA	11.7%	16.7%	5%
		Metastatic: *n* = 16		Uncemented: *n* = 5				
Current study	76	Primary: *n* = 76	63 (12–104)	Uncemented: *n* = 76	90.4%	2.6%	4.0%	4.0%

Abbreviation: NA, not available.

In this study, we identified younger age (< 30 years) as a significant risk factor, while the length of resection did not appear to impact survival. Younger patients undergoing endoprosthetic replacement may experience reduced implant survival due to their higher activity levels. Additionally, their longer life expectancy means the endoprosthesis must endure functional demands over a longer period, further increasing the risk of revision. It is noted that the resection length is often considered a potential risk factor for implant survival [36], but our findings did not identify it as a significant factor influencing failure.

### 4.3 Complications

Infection remains a significant complication following proximal femoral replacement, with reported infection rates ranging from 1.2% to 16.7% [11, 12]. In our study, the infection rate was 4.0%, which is slightly higher than the 1.2% reported by Bernthal et al. in a series of 86 patients using modular endoprostheses [11]. However, it is notably lower than the 16.7% reported by Senna et al. in a cohort of 60 patients [12]. The elevated infection rate in their study was likely associated with part of the patients having undergone prior surgeries, including curettage or internal fixation. Additionally, some factors related to the increased infection are highlighted in several studies, such as soft tissue disruption, large endoprosthetic surface areas, and the effects of adjuvant therapies, especially radiation [37, 38, 39].

Aseptic loosening is a common failure mode for endoprosthetic replacement following tumor resection. The debate regarding whether cemented or uncemented stems offer better endoprosthetic fixation remains ongoing [16, 40, 41]. Regarding patients with metastatic tumors, the shorter survival time may limit the ability to observe aseptic loosening. For example, Guzik et al. reported no cases of aseptic loosening in a cohort of 100 patients with metastatic disease who underwent cemented prosthesis replacement, with a follow‐up duration of 15.9 months [35]. In cohorts that include a mix of primary tumor patients, the reported incidence of aseptic loosening varies widely, ranging from 1% to 11.6% in the literature [11, 12, 30, 33]. Overall, the use of cemented stems appears to be more prevalent than uncemented ones [42]. However, large comparative cohort studies directly evaluating aseptic loosening between cemented and uncemented endoprostheses remain lacking. In our study, a low rate of aseptic loosening was observed to be 2.6%. In both of the observed cases, loosening occurred within 2 years postoperatively, suggesting early failure of bone‐implant integration. Several factors may contribute to this complication, including insufficient primary press‐fit fixation and micromotion at the interface leading to fibrous tissue interposition rather than osseous ingrowth. Nevertheless, good interface osseointegration of the uncemented stems was observed in the majority of patients. In our study, the low rate of aseptic loosening and good interface osseointegration observed can be attributed to the advanced design of the uncemented stem. Specifically, the uncemented stem features a unique short and curved shape, which better matches the anterior curvature of the femoral medullary cavity and provides improved anti‐rotation stability. This design could minimize stress shielding and enhance proximal loading, addressing limitations seen in traditional straight or extensively long stems [20, 43, 44]. Acetabular wear is a well‐recognized problem following bipolar hemiarthroplasties [45]. This condition is often associated with groin pain and can, in more severe cases, necessitate conversion to total hip arthroplasty (THA) due to progressive acetabular cartilage loss. In our study regarding proximal femur replacement following primary tumor resection, acetabular failure requiring revision was observed in 4.0% of cases, which is comparable to previous reports in the literature [11, 12, 30, 33]. However, it is noted that all patients who developed acetabular wear in our series were under 30 years of age. Similarly, Bernthal et al. observed that patients requiring THA conversion were younger (mean age 27.5 years) compared to their overall cohort (mean age 44.5 years) [11]. Younger patients may exert greater physical demands on the prosthesis, potentially accelerating acetabular wear. Further research into long‐term outcomes and the development of more durable materials could help mitigate the risk of acetabular failure in young patients undergoing hemiarthroplasty.

### 4.4 Future Perspectives on Endoprosthesis Design

To further improve outcomes and reduce complications after proximal femoral endoprosthetic replacement, future endoprosthetic designs should integrate strategies targeting failure modes. Incorporating bioactive factors or surface modifications that promote osteointegration may help reduce aseptic loosening. Antibacterial coatings or drug‐eluting surfaces could lower the risk of periprosthetic infection. Additionally, embedding localized anti‐tumor agents within the implant may offer prophylaxis against tumor recurrence. Such multifunctional designs that combine structural support with therapeutic capability represent a promising direction for next‐generation endoprostheses.

### 4.5 Limitations and Strengths

Our study is associated with several limitations. First, we lacked control groups for comparison, such as patients with metastatic bone tumors or those treated with cemented endoprostheses. The absence of these control groups limits our ability to directly compare the outcomes of modular uncemented endoprostheses with alternative reconstruction strategies. Second, although our analysis identified younger age (< 30 years) as a significant risk factor for reduced implant survival, this association may be confounded by underlying clinical variables. In our cohort, most younger patients were diagnosed with osteosarcoma, a malignancy typically treated with intensive chemotherapy. Chemotherapy can impair bone remodeling, delay soft tissue healing, and increase infection risk, all of which may compromise implant longevity. Therefore, future studies with larger cohorts and multivariate analyses are needed to clarify the respective impacts of age, chemotherapy, and tumor biology on prosthesis survival. Despite these limitations, our study possesses several notable strengths. First, it represents one of the largest single‐center cohorts focused exclusively on primary bone tumor patients undergoing proximal femoral replacement with modular uncemented endoprostheses. Second, all procedures were performed using the same implant system with consistent surgical techniques, which reduce device‐related variability. Third, the relatively long follow‐up duration (mean 63.4 months) allows for robust assessment of mid‐ to long‐term implant performance. Lastly, we employed detailed radiographic evaluation methods including tomosynthesis‐based osseointegration criteria, providing objective insights into implant fixation and bone‐implant interface behavior.

## 5 Conclusions

In conclusion, this study represents one of the largest cohorts of patients with primary bone tumors undergoing proximal femoral replacement. The findings underscore the favorable outcomes associated with modular uncemented endoprostheses, including a 5‐year endoprosthesis survival rate of 90.4% and a mean MSTS score of 25.6, reflecting excellent functional recovery and durability. Younger age was identified as a risk factor for reduced implant longevity, likely due to higher activity levels, while resection length did not appear to influence survival outcomes. The advanced design of the uncemented stem, which promotes good osseointegration and mechanical stability, likely contributed to the low rate of aseptic loosening (2.6%) observed in this cohort. These results suggest that modular uncemented systems are a reliable and durable solution for the reconstructive demands of proximal femoral replacement in patients with primary bone tumors.

## Author Contributions

**Chongqi Tu, Li Min, Minxun Lu:** conceptualization. **Zhuangzhuang Li, Yitian Wang, Taojun Gong:** methodology and data collection. **Zhuangzhuang Li, Yi Luo, Xuanhong He, Yong Zhou:** data analysis. **Zhuangzhuang Li, Yi Luo:** writing – original draft. **Minxun Lu, Li Min:** writing – review and editing. **Yitian Wang, Minxun Lu, Chongqi Tu:** supervision and project administration.

## Ethics Statement

This study was performed in accordance with the Declaration of Helsinki as revised in 2008 and was approved by the Ethics Committee of West China Hospital.

## Consent

All patients' parents signed the informed consent form before surgery and provided consent to publish and report individual clinical data.

## Conflicts of Interest

The authors declare no conflicts of interest.

## Data Availability

The data that support the findings of this study are available from the corresponding author upon reasonable request.
